# Assessment of Growth and Development in Children With Hepatitis B Positivity

**DOI:** 10.14740/gr628e

**Published:** 2014-12-27

**Authors:** Tugba Sari, Erdal Eren, Suda Tekin Koruk

**Affiliations:** a Department of Infectious Diseases and Clinical Microbiology, Harran University School of Medicine, 63100 Sanliurfa, Turkey; b Department of Pediatric Endocrinology, Harran University School of Medicine, 63100 Sanliurfa, Turkey

**Keywords:** Hepatitis B, Children, Body weight, Body height

## Abstract

**Background:**

Chronic infections and liver diseases may influence the growth and development of children by leading to malnutrition. In this study, demographic characteristics, anthropometric measurements and laboratory findings for children with hepatitis B positivity were analyzed.

**Methods:**

A total of 43 cases were admitted to our clinic between January 2012 and February 2013 and detected to have HBsAg positivity.

**Results:**

Malnutrition was detected in 11 cases (25.6%) and obesity in three cases (6.9%). Aspartate aminotransferase (AST) levels were significantly higher in malnourished patients compared to those without malnutrition. The weight to height was significantly higher in patients with positive HBeAg compared to children with negative HBeAg. We found that the weight standard deviation scores (SDS) ratios dropped as alanine aminotransferase (ALT) and AST levels increased and height SDS ratios decreased. In addition, body mass index (BMI) decreased as AST and alpha feto protein (AFP) values increased. While a significant relationship was not detected between insulin-like growth factor binding protein-3 (IGFBP-3) and insulin-like growth factor-1 (IGF-1) and ALT, a significantly negative correlation was detected between IGFBP-3 and IGF-1 and AST. We found a malnutrition rate of 25.6% in children with HBsAg positivity. We also found that weight and height SDS rates decreased as ALT and AST levels increased. In addition, we detected that BMI decreased as AST and AFP values increased.

**Conclusion:**

We consider that hepatic inflammation is the factor that affects growth. Monitoring of growth and development during follow-up of children who are detected to have HBsAg positivity would be beneficial to determine the mechanism and causes of growth retardation.

## Introduction

Children’s growth is a multifactorial process involving genetic and environmental factors. Infections, particularly of gastrointestinal origin, may lead to anorexia, reduction in energy and metabolic rate and malnutrition [1-3]. Malnutrition makes children susceptible to infections through a vicious cycle [4-10]. Chronic infections and liver diseases may influence the growth and development of children by leading to malnutrition [4-10]. Anorexia, impaired nutritional habits and impaired insulin-like growth factor (IGF) release are the most common causes of malnutrition in chronic liver diseases [5-8]. The hepatitis B virus (HBV) is an important health problem in our country and in the world, as it leads to chronic hepatitis, hepatic cirrhosis and hepatocellular carcinoma, in addition to acute hepatitis [11]. In this study, demographic characteristics, anthropometric measurements and laboratory findings for children with hepatitis B positivity were analyzed.

## Methods

This was a prospective cohort study implemented from January 2012 to February 2013 in Sanliurfa, Turkey. A total of 43 cases who were admitted to Infectious Diseases and Clinical Microbiology Clinic and detected to have HBsAg positivity, in whom acute viral hepatitis was excluded with serologic tests were randomly enrolled in the study. The height, weight and body mass index (BMI) of patients and the standard deviation scores (SDS) of these parameters were evaluated. Ethics committee approval was obtained for this study investigating the influence of HBsAg positivity on growth in children. The height and weight of all participants were measured. The weight measurement was done using a weighing scale sensitive to 100 g. The height measurement was done with 0.5 cm sensitivity. The individual percentile values of children were determined. BMI (kg/m^2^) was calculated. These values were compared with those determined by Neyzi et al [12] for Turkish children. Children with a weight to height value below 90% were accepted as malnourished, and those above 120% were accepted as obese [13]. Serum insulin-like growth factor-1 (IGF-1) and insulin-like growth factor binding protein-3 (IGFBP-3) were studied. A DSL-5600 Active^TM^ IGF-1 IRMA kit and a DSL-6600 Active^TM^ IGFBP-3 IRMA kit were used in a Berthold Lb 2111 12 detector gamma counter device for analysis (Diagnostic System Laboratories Inc., Webster, TX, USA). HBV DNA, alanine aminotransferase (ALT), aspartate aminotransferase (AST), gamma glutamyl transferase (GGT), IGF-1, IGFBP-3, total bilirubin, direct bilirubin, alpha feto protein (AFP) and HBeAg positivity were also examined. The normal laboratory values and methods are given in Table 1.

**Table 1:** Normal Values and Laboratory Procedures

Parameters	Cutoff points	Method
AST (U/L)		Colorimetric
1 - 9 years	15 - 55	
10 - 19 years	5 - 45	
ALT (U/L)		Colorimetric
1 - 19 years	5 - 45	
GGT (U/L)		Colorimetric
4 months - 10 years	5 - 32	
10 - 15 years	5 - 24	
ALP (U/L)		Colorimetric
< 15 years	350 - 1,000	
> 15 years	25 - 250	
Total bilirubin (mg/dL)	0.2 - 1.3	Colorimetric
AFP (ng/mL)	0 - 14	Chemiluminescence
IGF-1 (ng/mL)		Enzymatic-labeled chemiluminescent immunometric assay
0 - 4 years	49 - 171	
> 4 years	76 - 499	
IGFBP-3 (ng/mL)		Enzymatic-labeled chemiluminescent immunometric assay
5 - 10 years	1.1 - 7	
10 - 15 years	2.4 - 9.5	
HBV DNA (IU/mL)		Polymerase chain reaction
Positive	< 2,000	
Negative	> 2,000	
HAI score		Knodell scoring system
Minimal inflammation	1 - 4	
Mild-marked inflammation	> 4	

Patients were evaluated according to biopsy results, ultrasonography findings, HBV DNA negativity spontaneously or through treatment, ALT normalization and HBeAg seroconversion. Modified Knodell scoring was used for histopathologic staging in chronic patients [14]. Patients with ALT more than two-fold of normal values and HBV DNA > 2,000 IU/mL were accepted as chronic hepatitis B. Those whose HBeAg was positive and ALT level was normal but HBV DNA > 2,000 IU/mL were accepted as immune tolerant. Those whose HBeAg was negative and ALT level was normal and HBV DNA < 2,000 IU/mL were accepted as carriers [15]. Patients who had malabsorption, growth hormone deficiency and syndromes were excluded from the study. The SPSS 15.0 statistical package program was used for statistical analysis. Pearson’s correlation test, the Chi-square test and the *t*-test were used for data analysis. A P level of < 0.05 was accepted as statistically significant.

## Results

Of the patients enrolled in the study, 17 (39.5%) were girls, and the mean age of the patients was 10.81 ± 3.23 years; the mean age at the time of diagnosis was 7.27 ± 3.45 years. The mean duration of follow-up at our clinic was 35.16 ± 24.8 months. There was a family history of hepatitis B in 37 (86%) patients; hepatitis B positivity was detected in the mother in 25 (58.1%) patients and in at least one sibling in 28 (65.1%) patients. All patients had similar family properties with low socioeconomic status. The demographic characteristics of patients are given in Table 2.

**Table 2:** Features of Children Diagnosed With Hepatitis B

Age	
Mean age	10.81 ± 3.23
Age range	2 - 16.49
SDS for height	-0.29 ± 0.88 (-2.15 - 1.47)
SDS for weight	-0.30 ± 0.99 (-2.52 - 1.67)
SDS for BMI	-0.25 ± 1.25 (-4.83 - 1.89)
Gender	
Girl	17 (39.5%)
Boy	26 (60.5%)
Potential way of contamination	
Circumcision at home	1 (2.3%)
Operation	3 (7%)
Dental treatment	3 (7%)
Hospitalization	1 (2.3%)
Family history of hepatitis B	
Yes	37 (86%)
No	6 (14%)
Follow-up (month)	2 - 84
Mean	35.16 ± 24.8
Treated	6 (14%)
Lamivudine	5 (11.6%)
Adefovir	1 (2.3%)
Previously received interferon + lamivudine	1 (2.3%), maintenance with lamivudine
Not treated	37 (86%)

The height, weight and BMI of patients and the SDS of the parameters were analyzed. While the SDS for height was below -2 in one child with HBsAg positivity, the SDS for weight was below -2 in three.

HBeAg was positive in 29 of 43 participants (67.4%), and AntiHBe was positive in 14 (32.6%). Of the cases, 14 (42.6%) were carriers, 13 (30.2%) were chronic patients and 16 (37.2%) were immune tolerant. The laboratory findings of patients are given in Table 3.

**Table 3:** Laboratory Findings of Patients

	Mean ± SD
AST (U/L)	12 - 66 (35.2 ± 11.1)
ALT (U/L)	9 - 113 (37.6 ± 21)
GGT (U/L)	6 - 29 (11.7 ± 3.8)
ALP (U/L)	71 - 491 (259.27 ± 81.06)
Total bilirubin (mg/dL)	0.2 - 1.3 (0.22 ± 0.1)
Direct bilirubin (mg/dL)	0.1 - 0.5 (0.22 ± 0.1)
AFP (IU/mL)	0 - 5.4 (1.5 ± 1.14)
IGF-1 SDS (ng/mL)	-2.27 - 6.81 (0.5 ± 1.94)
IGFBP-3 SDS (ng/mL)	-3.83 - 2.59 (0.16 ± 1.40)

Malnutrition was detected in 11 cases (25.6%) and obesity in three cases (6.9%). Patients with and without malnutrition are compared in Table 4. While malnutrition was detected in 11 (n = 37) (29.7%) of the patients with a family history of hepatitis B, malnutrition was detected in none of the children without a family history of hepatitis B (n = 6). AST levels were significantly higher in malnourished patients compared to those without malnutrition (P = 0.004).

**Table 4:** Comparison of Mean Laboratory Findings of Children With or Without Malnutrition

	Malnutrition (+)	Malnutrition (-)	P value
AST (U/L)	41.09 ± 13.31	33.25 ± 9.75	0.04
ALT (U/L)	43.45 ± 31.59	35.59 ± 16.63	0.297
GGT (U/L)	10.72 ± 4.31	12.09 ± 3.68	0.316
HAI score	6*	5.6 ± 2.8	0.905
HBV DNA (IU/mL)	1 × 10^8^ ± 8 × 10^7^	7 × 10^7^ ± 6 × 10^7^	0.223
IGF-1 SDS (ng/mL)**	-0.87 ± 0.84	0.8 ± 2	0.031
IGFBP-3 SDS (ng/mL)**	-0.53 ± 0.93	0.35 ± 1.46	0.13

*Malnutrition was detected in only one child who underwent biopsy, and HAI was found to be 6. **IGF-1 level was studied in 32 patients, and IGFBP-3 level was studied in 33 patients.

ALP and GGT levels were found to increase as HBV DNA values increased (P = 0.02, P < 0.001). The HBV DNA and AST values of HBeAg-positive children were significantly greater (P < 0.001, P = 0.02). The ALT level was significantly higher in children with negative HBeAg (P = 0.007). The weight to height was significantly higher in patients with positive HBeAg compared to children with negative HBeAg (P = 0.03). We found that the weight SDS ratios dropped as ALT and AST levels increased (P = 0.048, P < 0.001) and height SDS ratios decreased (P = 0.015, P = 0.014). In addition, BMI decreased as AST and AFP values increased (P = 0.009, P = 0.001).

The IGF-1 level was studied in 32 patients, and IGFBP-3 was studied in 33 patients. The IGF-1 level was significantly lower in malnourished patients (P = 0.031). While a significant relationship was not detected between IGFBP-3 and IGF-1 and ALT, a significantly negative correlation was detected between IGFBP-3 and IGF-1 and AST (P = 0.01, P = 0.013).

Liver biopsy was performed in six (14%) patients. The results were as follows: histological activity index (HAI): 1 (n = 1), HAI: 5 (n = 1), HAI: 6 (n = 2), HAI: 8 (n = 2), fibrosis: 1 (n = 3), fibrosis: 2 (n = 1), fibrosis: 3 (n = 2). Malnutrition was detected in one of them (HAI = 6). HBV DNA became negative in two out of six patients who were treated (4.7%), ALT became normal in four (9.3%) and HBeAg seroconversion was detected in two (4.7%). In untreated patients, HBV DNA became negative spontaneously during follow-up in one patient (2.3%), ALT became normal in 10 (23.3%) and HBeAg seroconversion was seen in two (4.7%).

Hepatobiliary ultrasonography revealed normal findings in 40 children (93%), rough granular appearance in 2 (4.7%) and gallbladder stone in 1 (2.3%).

## Discussion

Vertical contamination from an infected mother to a child is the main source of infection in many endemic countries [16, 17]. HBV infection causes infection mainly via non-parenteral routes in moderately endemic countries such as our country [18, 19]. In our study, there was a history of hepatitis B in the families of 37 patients (86%); HBsAg positivity was detected in the mother in 25 of them (58.1%) and in at least one sibling in 28 (65.1%).

Food intake is reduced and food loss is increased in all infectious diseases due to impaired intestinal absorption and increased intestinal excretion [10]. Reduced calorie intake, malabsorption and chronic liver diseases affect IGF production from the liver, or inflammatory mediators may affect growth [20].

In addition, the elevation of inflammatory cytokines may affect anorexia [10, 21, 22]. Another important mechanism is catabolic processes, which continue during infections, negatively affecting growth [10]. The immune stimulant effect of subclinical infections may also lead to malnutrition in developing countries [10, 23]. Growth retardation is among the rare outcomes of chronic hepatitis B [4-10]. Regulation of the liver metabolism plays an important role in food homeostasis and absorption [5-8].

Chronic infections and chronic liver diseases are known to lead to malnutrition through different mechanisms [4-10]. However, growth retardation has been rarely reported in chronic hepatitis B [24, 25]. Growth retardation has been detected in children who were diagnosed with chronic hepatitis B and received interferon treatment [26, 27]. A malnutrition rate of 21% was found in another study carried out with 3,152 preschool children [28]. In the study of Kuloglu et al [29], hepatitis B was found not to affect growth and development. We found a malnutrition rate of 25.6% in children with HBsAg positivity. We also found that weight and height SDS rates decreased as ALT and AST levels increased. In addition, we detected that BMI decreased as AST and AFP values increased. We consider that hepatic inflammation is the factor that affects growth.

Children’s living in rural areas, having families with low education level, low socioeconomic level, poor hygiene and having many siblings negatively affect their nutritional status. HBV seroprevalence is high in rural areas that have a low socioeconomic level [28].

Of our patients, 32.6% were inactive carriers, 30.2% were chronic hepatitis B patients and 37.2% were in an immune tolerant phase. Most of the immune tolerant children had been affected via the perinatal route. T cells’ likelihood of infecting hepatocytes is lower, as their immune system has not been fully developed. Therefore, these children pass to an immune competent phase after decades as they develop normal transaminase levels [20, 30-32].

HIV positivity was shown to affect growth and development negatively in correlation with viral load [33]. In our study, HBV load was not found to be correlated with growth and development.

Growth hormone’s anabolic and growth accelerating effects are realized through IGF-1 and IGFBP-3. IGF-1 is mainly released from liver and peripheral tissues, and IGF-1 level measurement may be used to determine growth hormone deficiency. Serum IGF-1 and IGFBP-3 levels are affected by chronological age, sexual maturity and nutritional status [34, 35]. In the study of Colakoglu et al [36], IGF-1 and IGDBP-3 levels were found to be significantly lower in 42 cirrhotic patients compared to non-cirrhotic ones. We detected that IGF-1 and IGFBP-3 values decreased as AST levels increased.

We found that HBV infection affected the development of children. Generally, elevated AST levels were effective, in development of children and we aimed to emphasize that this infection should be kept in mind while researching developmental anomalies. Monitoring of growth and development during follow-up of children who are detected to have HBsAg positivity would be beneficial to determine the mechanism and causes of growth retardation.

## Author Note

The present address of Tugba Sari is Infectious Diseases and Clinical Microbiology Department, Buldan Chest Diseases Hospital, 20400 Denizli, Turkey.

## References

[1] Zenel JA (1997). Failure to thrive: a general pediatrician's perspective. Pediatr Rev.

[2] Marcovitch H (1994). Failure to thrive. BMJ.

[3] Roy CC, Silverman A, Alagille D, Roy CC, Silverman A, Alagille D (1995). Symptoms. Pediatric clinical gastroenterology.

[4] Doherty C, Reilly J, Paterson W, Walker WA, Goulet O, Kleinman RE (2004). Growth failure. Pediatric gastrointestinal disease: pathophysiology, diagnosis, management.

[5] Shepherd RW, Ramm GA, Walker WA, Goulet O, Kleinman RE, Sherman PM, Schneider BL, Sanderson IR (2004). Fibrogenesis and cirrhosis. Pediatric gastrointestinal disease: pathophysiology, diagnosis, management.

[6] Mager DR, Wykes LJ, Roberts EA, Ball RO, Pencharz PB (2006). Mild-to-moderate chronic cholestatic liver disease increases leucine oxidation in children. J Nutr.

[7] Mager DR, Wykes LJ, Roberts EA, Ball RO, Pencharz PB (2006). Branched-chain amino acid needs in children with mild-to-moderate chronic cholestatic liver disease. J Nutr.

[8] Bavdekar A, Bhave S, Pandit A (2002). Nutrition management in chronic liver disease. Indian J Pediatr.

[9] Tsiaousi ET, Hatzitolios AI, Trygonis SK, Savopoulos CG (2008). Malnutrition in end stage liver disease: recommendations and nutritional support. J Gastroenterol Hepatol.

[10] Bhutta ZA (2006). Effect of infections and environmental factors on growth and nutritional status in developing countries. J Pediatr Gastroenterol Nutr.

[11] Tasyaran MA, Kilicturgay K, Badur S (2013). The epidemiology of HBV infection. Journal of Viral hepatitis. Istanbul: The association of Viral hepatitis.

[12] Neyzi O, Gunoz H, Furman A (2008). Body weight, height, head circumference and body mass index reference values for Turkish children. Journal of children health and diseases.

[13] Waterlow JC (1972). Classification and definition of protein-calorie malnutrition. Br Med J.

[14] Ishak K, Baptista A, Bianchi L, Callea F, De Groote J, Gudat F, Denk H (1995). Histological grading and staging of chronic hepatitis. J Hepatol.

[15] (2012). EASL clinical practice guidelines: Management of chronic hepatitis B virus infection. J Hepatol.

[16] http://www.who.int/immunization_monitoring/Global_Immunization_Data.pdf

[17] Chan OK, Lao TT, Suen SS, Leung TY (2012). Deficient knowledge on hepatitis B infection in pregnant women and prevalence of hepatitis B surface antigen carriage in an endemic area: a review. Hepat Res Treat.

[18] Ersoy Y, Sonmez E, Cetin C (1997). Hepatitis B virus transmission in the family. Journal of Turgut Ozal medical center.

[19] Ustun C, Basuguy E, Deveci U Seroprevalence of hepatitis B and hepatitis C in children admitted to pediatric surgery policlinic. Online journal of Nobel medicus.

[20] Gerner P, Horning A, Kathemann S, Willuweit K, Wirth S (2012). Growth abnormalities in children with chronic hepatitis B or C. Adv Virol.

[21] Bhutta ZA, Mansoorali N, Hussain R (1997). Plasma cytokines in paediatric typhoidal salmonellosis: correlation with clinical course and outcome. J Infect.

[22] Campbell DI, Elia M, Lunn PG (2003). Growth faltering in rural Gambian infants is associated with impaired small intestinal barrier function, leading to endotoxemia and systemic inflammation. J Nutr.

[23] Solomons NW, Mazariegos M, Brown KH, Klasing K (1993). The underprivileged, developing country child: environmental contamination and growth failure revisited. Nutr Rev.

[24] Polito C, La Manna A, Cartiglia ML, Bonomo G, Del Gado R (1991). Normal growth of children with HBsAg positive chronic active hepatitis (CAH). Acta Paediatr Scand.

[25] Vegnente A, Guida S, Di Costanzo V, Fusco C, Iorio R, Toscano P (1992). Nutritional status and growth in children with chronic hepatitis B. J Pediatr Gastroenterol Nutr.

[26] Jara P, Bortolotti F (1999). Interferon-alpha treatment of chronic hepatitis B in childhood: a consensus advice based on experience in European children. J Pediatr Gastroenterol Nutr.

[27] Comanor L, Minor J, Conjeevaram HS, Roberts EA, Alvarez F, Bern EM, Goyens P (2001). Impact of chronic hepatitis B and interferon-alpha therapy on growth of children. J Viral Hepat.

[28] Tuncbilek E, Unalan T, Coskun T (1996). Indicators of nutritional status in Turkish preschool children: results of Turkish Demographic and Health Survey 1993. J Trop Pediatr.

[29] Kuloglu Z, Kansu A, Demirceken F, Arici ZS, Berberoglu M, Ocal G, Girgin N (2007). The influence of interferon-alpha and combination interferon-alpha and lamivudine therapy on height and weight in children with chronic hepatitis B infection. J Pediatr Endocrinol Metab.

[30] Milich DR, Jones JE, Hughes JL, Price J, Raney AK, McLachlan A (1990). Is a function of the secreted hepatitis B e antigen to induce immunologic tolerance in utero?. Proc Natl Acad Sci U S A.

[31] Chen MT, Billaud JN, Sallberg M, Guidotti LG, Chisari FV, Jones J, Hughes J (2004). A function of the hepatitis B virus precore protein is to regulate the immune response to the core antigen. Proc Natl Acad Sci U S A.

[32] Milich D, Liang TJ (2003). Exploring the biological basis of hepatitis B e antigen in hepatitis B virus infection. Hepatology.

[33] Hilgartner MW, Donfield SM, Lynn HS, Hoots WK, Gomperts ED, Daar ES, Chernoff D (2001). The effect of plasma human immunodeficiency virus RNA and CD4(+) T lymphocytes on growth measurements of hemophilic boys and adolescents. Pediatrics.

[34] Bar A, Tarasiuk A, Segev Y, Phillip M, Tal A (1999). The effect of adenotonsillectomy on serum insulin-like growth factor-I and growth in children with obstructive sleep apnea syndrome. J Pediatr.

[35] Juul A, Bang P, Hertel NT, Main K, Dalgaard P, Jorgensen K, Muller J (1994). Serum insulin-like growth factor-I in 1030 healthy children, adolescents, and adults: relation to age, sex, stage of puberty, testicular size, and body mass index. J Clin Endocrinol Metab.

[36] Colakoglu O, Taskiran B, Colakoglu G, Kizildag S, Ari Ozcan F, Unsal B (2007). Serum insulin like growth factor-1 (IGF-1) and insulin like growth factor binding protein-3 (IGFBP-3) levels in liver cirrhosis. Turk J Gastroenterol.

